# Inconsistencia en la implementación efectiva de planes de cuidado para las supervivientes de cáncer de mama

**DOI:** 10.1016/j.aprim.2025.103318

**Published:** 2025-07-29

**Authors:** Lara López Cornudella, Ana Lozano

**Affiliations:** a Facultat de Medicina i Ciències de la Salut, Universitat de Barcelona, Barcelona, España; b Servei de Medicina Interna, Hospital Universitari Sagrat Cor, Grupo Quirónsalud, Barcelona, España

*Sr. Editor,*

El reciente artículo titulado «Factores determinantes en la calidad de vida de las mujeres supervivientes de cáncer de mama»^1^ pone en evidencia una realidad que no debe ser ignorada: la importancia de la atención integral a mujeres que han superado el cáncer de mama, porque este no termina con la última sesión de tratamiento. A pesar de las numerosas directrices y recomendaciones, la implementación efectiva de planes de cuidado para las supervivientes sigue siendo inconsistente a nivel mundial.

En una revisión exploratoria, se valoró la efectividad de diversas herramientas interactivas de toma de decisiones que se han introducido recientemente en la práctica clínica para optimizar la atención a la supervivencia de cáncer de mama^2^. Se propone el uso de plataformas digitales basadas en evidencia como alternativa de práctica médica respaldada por dispositivos móviles, monitorización de pacientes o asistentes digitales personales. Siguiendo el estándar internacional de ayuda para la toma de decisiones del paciente –IPDAS–^2^, se obtuvieron resultados satisfactorios en varias variables que se engloban en la calidad de vida general de las mujeres supervivientes. Podría considerarse una buena opción para potenciar la atención integral a estas mujeres y solventar el problema que el artículo expone.

Por otro lado, el artículo cita el miedo a la recurrencia como el segundo dominio más relevante para las supervivientes de cáncer de mama. Niveles elevados de este miedo se asocian con un aumento de síntomas depresivos, ansiedad, estrés postraumático y trastornos psiquiátricos, e incluso pueden conducir al incumplimiento de las recomendaciones de atención de seguimiento y a demoras en la solicitud de atención médica para síntomas potenciales.

Un estudio transversal realizado en China resalta la importancia de comprender y gestionar el miedo a la recurrencia. La investigación identifica 3 factores determinantes asociados a este miedo: la situación laboral, el modo de afrontamiento médico y el apoyo social^3^. Para aliviar esta preocupación se enfatiza en la necesidad de que las pacientes adopten un enfoque proactivo y confortativo, brindándoles estrategias de afrontamiento efectivas y abordando patrones de evitación o resignación. Además, se subraya la importancia del apoyo emocional por parte de familiares y amigos, así como del apoyo social objetivo, instrumental y evaluativo, para ayudarles a mitigar el impacto del miedo a la recurrencia en su bienestar. El seguimiento psicológico individualizado se considera un pilar fundamental determinante de la calidad de vida de estas mujeres.

En conclusión, la calidad de vida de las supervivientes de cáncer de mama puede verse comprometida por múltiples factores incluso después de la remisión de la enfermedad. Dada la alta incidencia de esta enfermedad, resulta fundamental abordar este problema de manera integral para optimizar el bienestar de un gran número de mujeres. Sin embargo, surge la pregunta clave: ¿cómo podemos traducir este conocimiento en estrategias efectivas dentro de la práctica clínica?
